# Synthesis, Properties, and Biodegradation of Sequential Poly(Ester Amide)s Containing γ-Aminobutyric Acid

**DOI:** 10.3390/ijms21103674

**Published:** 2020-05-23

**Authors:** Yuushou Nakayama, Kazumasa Watanabe, Ryo Tanaka, Takeshi Shiono, Norioki Kawasaki, Naoko Yamano, Atsuyoshi Nakayama

**Affiliations:** 1 Department of Applied Chemistry, Graduate School of Engineering, Hiroshima University, 1-4-1 Kagamiyama, Higashi-Hiroshima, Hiroshima 739-8527, Japan; m173038@hiroshima-u.ac.jp (K.W.); rytanaka@hiroshima-u.ac.jp (R.T.); tshiono@hiroshima-u.ac.jp (T.S.); 2 Biomedical Research Institute, National Institute of Advanced Industrial Science and Technology (AIST), 1-8-31 Midorigaoka, Ikeda, Osaka 563-8577, Japan; n-kawasaki@aist.go.jp (N.K.); yamano.naoko@aist.go.jp (N.Y.); a.nakayama@aist.go.jp (A.N.)

**Keywords:** poly(ester amide)s, γ-aminobutyric acid, thermal properties, biodegradation, seawater, activated sludge

## Abstract

Poly(ester amide)s are attracting attention because they potentially have excellent thermal and mechanical properties as well as biodegradability. In this study, we synthesized a series of novel poly(ester amide)s by introducing γ-aminobutyric acid (GABA) regularly into polyesters, and investigated their properties and biodegradabilities. GABA is the monomer unit of biodegradable polyamide 4 (PA4). The new poly(ester amide)s were synthesized from the reaction of ammonium tosylate derivatives of alkylene bis(γ-aminobutylate) and *p*-nitrophenyl esters of dicarboxylic acids. All the obtained polymers showed relatively high melting temperatures (*T*_m_). Their thermal decomposition temperatures were improved in comparison with that of PA4 and higher enough than their *T*_m_. The poly(ester amide)s exhibited higher biodegradability in seawater than the corresponding homopolyesters. Their biodegradabilities in activated sludge were also studied.

## 1. Introduction

Biodegradable polymers are attracting attention due to recent environmental problems caused by wasted polymer materials [1,2,3,4]. Bacterial aliphatic polyesters such as poly(3-hydroxyalkanoate)s (PHAs) and synthetic polyesters such as poly(l-lactic acid) (PLLA), poly(glycolic acid) (PGA), poly(ε-caprolactone) (PCL), and poly(butylene succinate) (PBS) are representative biodegradable polymers. In recent years, marine pollution by microplastics has become a worldwide issue [5,6,7]. Therefore, the polymers biodegradable in seawater should be useful for the applications where the complete recovery of the materials is difficult. Among the so-called biodegradable polymers, ones degradable in seawater are limited. PHAs and PGA are the representative examples reported to be biodegradable in seawater [8,9,10]. In contrast, aromatic polyesters such as poly(ethylene terephthalate) (PET) are not recognized as biodegradable polymers, although some microorganisms were reported to degrade PET [11]. We have recently reported a series of aliphatic-aromatic copolyesters with periodic sequences, where the copolyesters with glycolate units, poly((ethylene diglycolate) terephthalate), exhibited biodegradability in seawater, while the corresponding copolyester with l-lactate was hardly degradable [12]. These results could be related to high and low biodegradability of PGA [13] and PLLA [10,14,15,16] in seawater, respectively.

On the other hand, polyamides (nylons) such as polyamide 6 (6-nylon) are also widely used for their high water absorption, crystallinity, chemical resistance, high melting point, strength, and flexibility derived from intra- and intermolecular hydrogen bond of amide groups. However, the commercially available polyamides are made from fossil resources and hardly biodegradable. In contrast, polyamide 4 (PA4) can be obtained from bio-based γ-aminobutyric acid (GABA), which can be produced by fermentation of glutamic acid [17]. PA4 is also reported to be biodegradable in various media including seawater [18,19,20,21,22,23]. However, PA4 has a disadvantage of relatively low thermal stability, which can be somewhat improved by end-group modification [24].

Poly(ester amide)s are polymers having both ester and amide bonds in their polymer main chains [25,26,27,28,29,30,31,32,33,34]. Poly(ester amide)s with aliphatic ester moieties can possess both biodegradability for aliphatic ester bonds and excellent physical properties due to the amide bonds. Poly(ester amide)s with various monomer combinations have been synthesized and investigated for their thermal, mechanical properties and degradability. Bayer has commercialized poly(ester amide) (BAK 1095) that consists of 1,4-butanediol, adipic acid, and ε-caprolactam, which possesses complete biodegradability as well as excellent thermal and mechanical properties and processability [35]. Several poly(ester amides) containing α-amino acids have been studied due to their enzymatic degradability [30,33,36].

As PA4 has been reported to be biodegradable in seawater [22,37], we expected that the poly(ester amide)s containing GABA units could also be the polymers biodegradable in seawater. In addition [38], the copolymers with regular sequences such as alternating and periodic copolymers could have some specific properties such as higher melting temperature (*T*_m_) and/or glass-transition temperature (*T*_g_) not expected from those of the corresponding homopolymers [39], in comparison with the corresponding random copolymers [12,25,34,38]. In this study, we synthesized a series of new sequential poly(ester amide)s containing GABA unit and investigated their thermal properties and biodegradabilities in seawater and in activated sludge.

## 2. Results

### 2.1. Synthesis of the Sequential Poly(Ester Amide)s

**Scheme 1:**
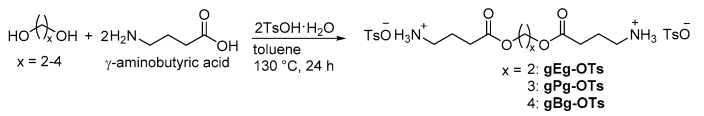
Synthesis of tosylates of alkylene γ-aminobutyrates.

**Scheme 2:**
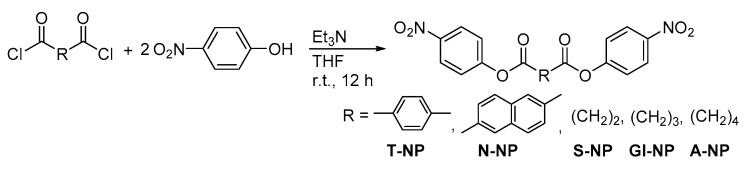
Synthesis of *p*-nitrophenyl esters of dicarboxylic acids.

In this study, the sequential poly(ester amide)s containing GABA unit were synthesized in a similar procedure for those containing α-amino acids [30,40]. For this purpose, tosylates of alkylene bis(γ-aminobutyrate)s and *p*-nitrophenyl esters of dicarboxylic acids were prepared from the reactions shown in Scheme 1 and Scheme 2, respectively. The 2:2:1 reaction of GABA, *p*-toluenesulfonic acid monohydrate (TsOH·H_2_O), and alkanediol in toluene gave the corresponding tosylates of alkylene bis(γ-aminobutyrate)s, gEg-OTs (alkylene = ethylene), gPg-OTs (alkylene = trimethylene), and gBg-OTs (alkylene = tetramethylene) in good yields after recrystallization from ethanol. On the other hand, the chloride derivatives of dicarboxylic acids such as terephthalic acid, 2,6-naphthalenedicarboxylic acid, succinic acid, glutaric acid, and adipic acid were reacted with *p*-nitrophenol in the presence of Et_3_N to produce the corresponding *p*-nitrophenyl esters, T-NP, N-NP, S-NP, Gl-NP, and A-NP, respectively [41,42,43,44].

**Scheme 3:**
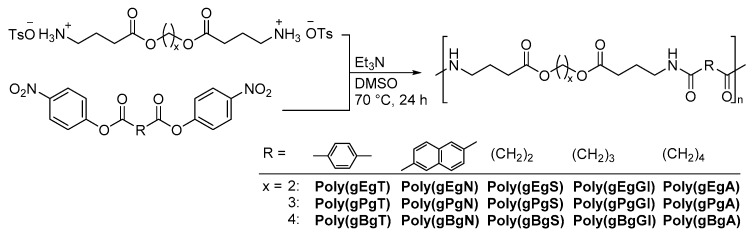
Polycondensation of the tosylates and the *p*-nitrophenyl esters to produce sequential poly(ester amide)s.

Polycondensation of the tosylates of alkylene bis(γ-aminobutyrate)s and the *p*-nitrophenyl esters of dicarboxylic acids in dimethyl sulfoxide (DMSO) was performed to produce sequential poly(ester amide)s (Scheme 3, Table 1). The poly(ester amide)s were obtained in good yields, due to the *p*-nitrophenol moiety acting as a good leaving group to promote the polymerization. The obtained poly(ester amide)s were poorly soluble in common organic solvents (tetrahydrofuran (THF), CHCl_3_), so 1,1,1,3,3,3-hexafluoro-2-propanol (HFIP) was used as an eluent in gel permeation chromatography (GPC) analysis calibrated with standard poly(methyl methacrylate)s. Their molecular weights were *M*_n_ = 2600–7600 g/mol. The polymerization reactions using different solvent such as tetrachloroethane, dimethylformamide, and *N*-methylpyrrolidone or longer reaction time (48 h) were also tested, however, the polymer yields and molecular weights were not improved.

**Figure 1:**
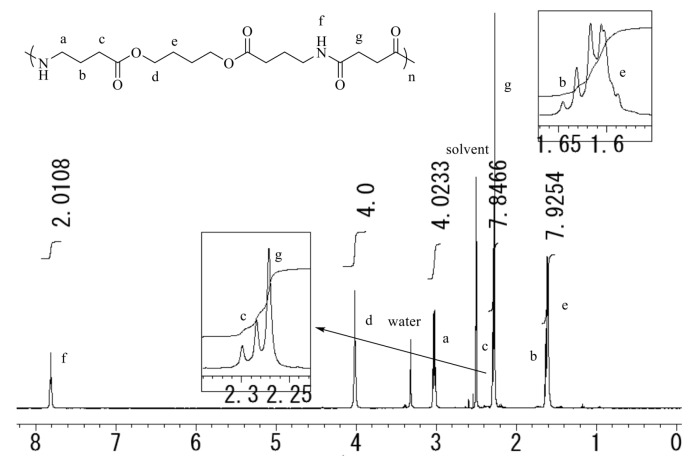
^1^H NMR spectrum of poly(gBgS) (DMSO-*d*_6_, 500 MHz, R.T.).

Figure 1 shows the ^1^H NMR spectrum of the obtained poly((butylene bis(γ-aminobutyrate)) succinate) (poly(gBgS)) for example. The observed seven signals can be assigned to the protons of the expected repeating unit, indicating the successful formation of the target poly(ester amide) with periodic sequence.

### 2.2. Thermal Properties of the Poly(Ester Amide)s Containing GABA Unit

We performed the differential scanning calorimetry (DSC) analysis of the obtained poly(ester amides)s to reveal their thermal properties, and the results are summarized in Table 2. All the polymers exhibited multiple melting transitions indicating their multiple crystal structures (Figure S19). Aromatic poly(ester amide)s containing terephthalate (entries 1–3) or 2,6-naphthalenedicarboxylate (entries 4–6) units exhibited relatively high *T*_m_ over 200 °C. Aliphatic poly(ester amide)s possessed *T*_m_ from 111 to 181 °C. Among them, the *T*_m_ values of glutarate polymers were lower than those of succinate and adipate polymers. Similar odd-even effects are known for simple aliphatic polyesters [45,46]. The poly(ester amide)s exhibited *T*_g_ from 71 to 103 °C.

**Figure 2:**
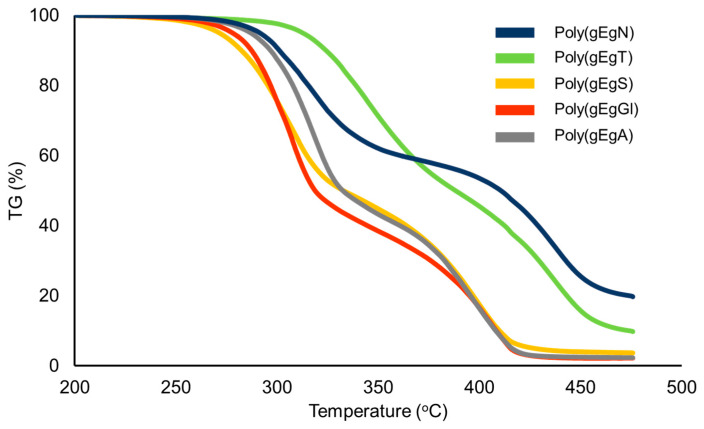
Thermogravimetric analysis (TG) curves of the selected poly(ester amide)s with gEg components.

The thermal degradation behaviors of the poly(ester amide)s were measured by thermogravimetric analysis (TG). The TG profiles of the poly(ester amide)s with gEg moiety are shown in Figure 2 for example, and those of others are included in the Supplementary Materials. All the poly(ester amide)s exhibited two stage thermal degradation behaviors. The degradation temperatures at 5% weight loss (*T*_d5_) and 10% weight loss (*T*_d10_) are included in Table 2.

### 2.3. Biodegradation of the Poly(Ester Amide)s Containing GABA Unit

Biodegradation tests were carried out in seawater and in activated sludge using the selected samples of the obtained poly(ester amide)s at 27 °C for one month. The biodegradation was monitored by the amount of the consumed oxygen. Based on the theoretical value of the amount of oxygen required for complete oxidation, which was calculated from the structure of each poly(ester amide), the degree of biodegradation was determined. A representative biodegradable polymer, poly(3-hydroxybutyrate) (PHB), was used as a reference sample.

**Figure 3:**
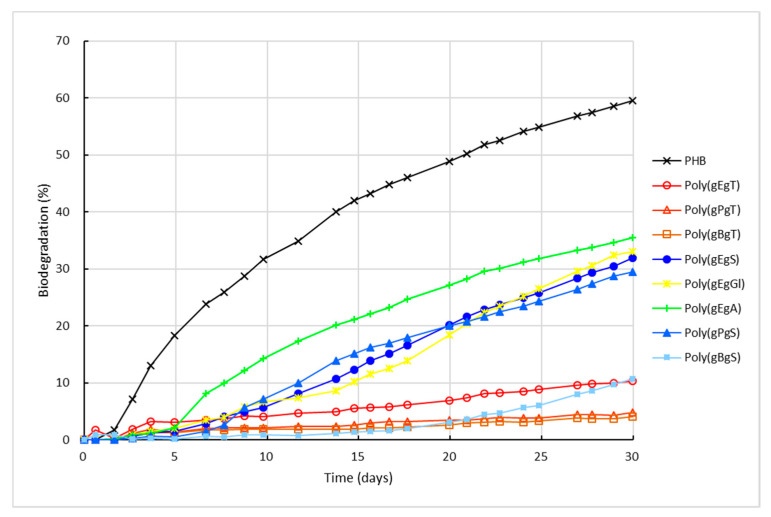
Biodegradation of the poly(ester amide)s in seawater.

The results of the biodegradation of the poly(ester amide)s in seawater are summarized in Figure 3. The poly(ester amide)s having ethylene glycol and aliphatic dicarboxylic acid units showed a biodegradation of ca. 30% after 30 days, similarly to PA4 homopolyer [37]. Poly(gPgS) with a C3 diol and succinate units also showed similar biodegradation to that of poly(gEgS). The biodegradation of poly(gBgS) and aromatic poly(ester amide)s were slower than those of the other poly(ester amide)s.

**Figure 4:**
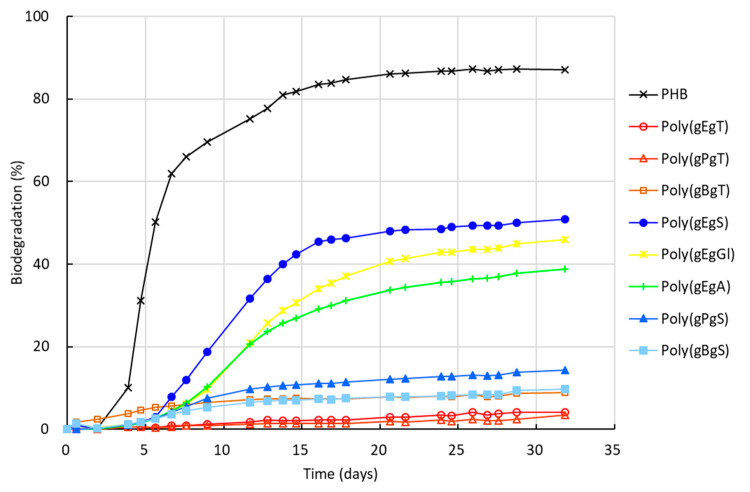
Biodegradation of the poly(ester amide)s in activated sludge.

Figure 4 shows the results of the biodegradation tests of the poly(ester amide)s in activated sludge. Although the biodegradation of the poly(ester amide)s were slower than that of PHB, the poly(ester amide)s with ethylene glycol unit were degraded significantly, as is the case in seawater. The other poly(ester amide)s were considerably less degraded than the aliphatic poly(ester amide)s with ethylene glycol units.

## 3. Discussion

We examined several synthetic methods to synthesize poly((ethylene bis(γ-aminobutyrate)) terephthalate) (poly(gEgT)). At first, we tried to synthesize poly(gEgT) by the reaction of ethylene bis(γ-aminobutyrate) (gEg) and terephthaloyl chloride. However, the purification of gEg was difficult because of its low stability, which resulted in low polymer yield and low molecular weight of the product. We also tested the polycondensation of dimethyl 4,4′-(terephthaloylbis(azanediyl))dibutyrate with ethylene glycol, but a significant amount of GABA component was lost during the reaction, possibly due to the formation of 2-pyrolidone. The solution polycondensation of the tosylates of alkylene γ-aminobutyrates and *p*-nitrophenyl esters of dicarboxylic acids resulted in better yields and structural regularities of the obtained poly(ester amide)s, possibly due to high reactivities of the reactants at relatively low temperature. The molecular weights of the obtained poly(ester amide)s still remained low to middle, which could be attributed to the purity of the reactants and/or relatively low solubility of the resulting polymers. The aliphatic poly(ester amide)s tended to have slightly higher molecular weights than those with aromatic components, this could come from the better solubility of the former.

The poly(ester amide)s with GABA showed multiple melting transitions, and a similar behavior was observed for other reported poly(ester amide)s [26,36]. This could be related to the liquid crystalline property, although not investigated in this study [26]. The *T*_m_ over 200 °C of the aromatic poly(ester amide)s are lower than those of the corresponding homopolyesters and PA4 but significantly higher than those of aliphatic poly(ester amide)s. The *T*_m_ values of the aliphatic poly(ester amide)s are higher than that of PBS (≈113 °C), which has the highest melting point among the polyesters of type (O(CH_2_)_x_OCO(CH_2_)_y_CO)_n_ (x = 2–4, y = 2–4) [47]. These results demonstrated that the sequential introduction of GABA units into aliphatic polyesters effectively enhance their *T*_m_ values.

**Figure 5:**
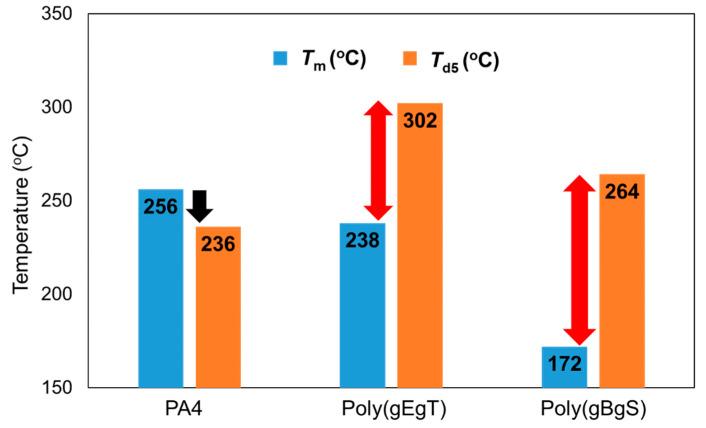
Comparison of melting and decomposition temperatures for the poly(ester amide)s. Black arrow: *T*_m_ > *T*_d5_. Red arrow: *T*_m_ < *T*_d5_.

The poly(ester amide)s showed two stage decomposition behavior in TG. Similar two step thermal degradation behaviors were observed for some reported poly(ester amide)s, where the first degradation step was assigned to the ester bond cleavage [34,49,50]. All the poly(ester amide)s showed higher *T*_d5_ values than that of PA4 [24] as indicated in Table 2. The *T*_m_ and *T*_d5_ values of the poly(gEgT), poly(gBgS), and PA4 are shown for example in Figure 5. PA4 has a higher *T*_m_ than its *T*_d5_. Therefore, PA4 has the difficulty in its thermal molding. In sharp contrast, *T*_d5_ values of the poly(gEgT) and poly(gBgS) are higher enough than their *T*_m_, indicating their excellent thermoformability.

**Figure 6:**
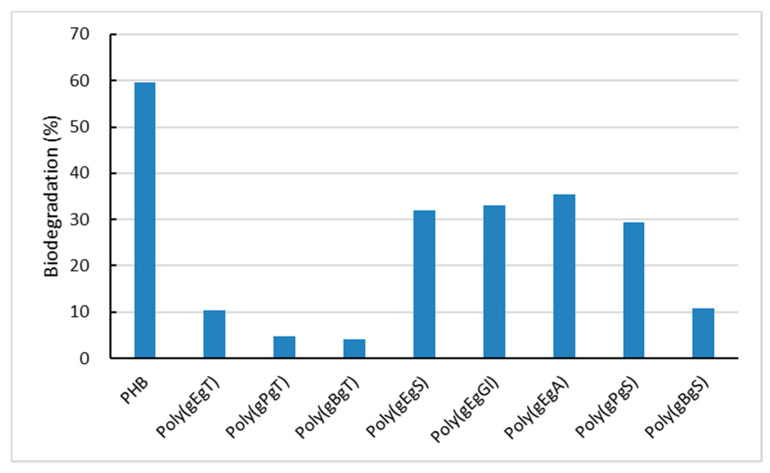
Biodegradation of the poly(ester amide)s in seawater after 30 days.

The observed biodegradation of the poly(ester amide)s in seawater after 30 days are summarized in Figure 6. The biodegradation of poly(ethylene succinate) (PES) and PBS in seawater was reported to be slow, where only 1–3% of those polymers were degraded after 28 days [10,51]. PET is hardly degradable in seawater over a year [9]. In contrast, biodegradation of poly(gEgS), poly(gBgS), and poly(gEgT) reached 30%, 10%, and 10%, respectively, although the conditions of these degradation tests are not completely the same as the cited references. These data suggest that the presence of GABA moiety in repeating units can enhance the biodegradability of the polymers in seawater, although rather low molecular weights of the poly(ester amide)s could somewhat contribute to their biodegradation, especially for poly(gEgT) (*M*_n_ = only 2600). The biodegradation of poly(gBgS) was much slower than those of poly(gEgS) and poly(gPgS), possibly due to the lower hydrophilicity of poly(gBgS). On the other hand, poly(gEgS), poly(gEgGl), and poly(gEgA) exhibited similar biodegradability. It is noteworthy that the biodegradation of poly(gEgA) was considerably faster than that of poly(gBgS) with the same composition. These results imply that the shorter diol unit is more important than the shorter dicarboxylate unit for faster biodegradation of the aliphatic poly(ester amide)s in seawater, although lower molecular weight of poly(gEgGl) and poly(gEgA) could also enhance their biodegradation. We assume that the ester bonds between diol and GABA units could be preferentially hydrolyzed rather than the amide bonds between GABA and dicarboxylate in the biodegradation of the poly(ester amide)s. This could explain the reason why the structure of the diol unit of the poly(ester amide)s has a critical effect on their biodegradation.

**Figure 7:**
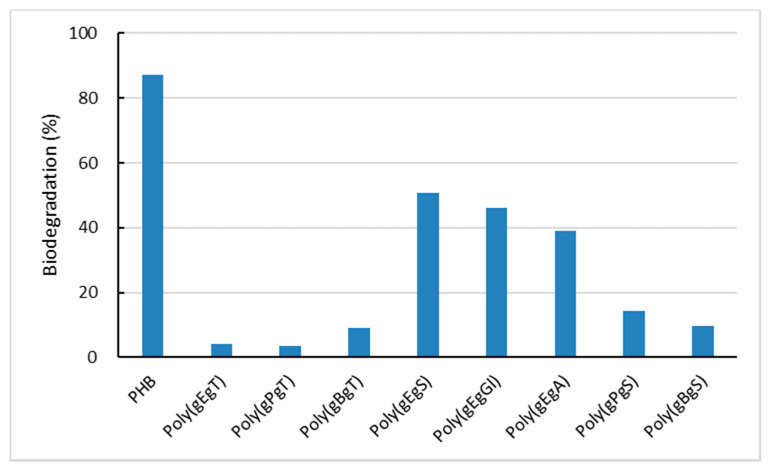
Biodegradation of the poly(ester amide)s in activated sludge after 32 days.

The observed biodegradation of the poly(ester amide)s in activated sludge after 32 days are summarized in Figure 7. As is the case in seawater, aliphatic poly(ester amide)s with ethylene glycol units were clearly more biodegradable than other poly(ester amide)s. Poly(gPgS) was less degradable in activated sludge than in seawater. The degradability of poly(gEgS) seems to be similar to those reported for PES [52] and PA4 [19], while that of poly(gBgS) seems to be lower than that of PBS [53], although the conditions are not the same.

## 4. Materials and Methods

### 4.1. Chemicals

All organic solvents were purified by distillation under nitrogen prior to use. Ethylene glycol (EG), 1,3-propanediol (PDO), 1,4-butanediol (BDO), and triethylamine (Et_3_N) (Tokyo Chemical Industry Co, Tokyo, Japan) were distilled under vacuum and stored on molecular sieves 4A. *p*-Toluenesulfonic acid monohydrate (TsOH·H_2_O), thionyl chloride (SOCl_2_), ethyl acetate (EtOAc), γ-aminobutyric acid (GABA), terephthaloyl chloride (TC), succinyl chloride (SC), glutaryl chloride (GC), adipoyl chloride (AC), 2,6-naphthalenedicarboxylic acid (NA), and *p*-nitrophenol were purchased from Tokyo Chemical Industry Co. Bis(*p*-nitrophenyl) terephthalate (T-NP) [41], bis(*p*-nitrophenyl) 2,6-naphthalenedicarboxylate (N-NP) [42], bis(*p*-nitrophenyl) succinate (S-NP) [41], bis(*p*-nitrophenyl) glutarate (G-NP) [43], and bis(*p*-nitrophenyl) adipate (A-NP) [44], were prepared according to the literature.

### 4.2. Measurements

^1^H NMR spectra were recorded on a Varian System 500 spectrometer (Agilent, Santa Clara, CA, USA) (500 MHz for ^1^H nucleus). Chemical shifts of ^1^H NMR spectra were calibrated by using residual proton in CDCl_3_ (δ = 7.26 ppm) and in DMSO-*d*_6_ (δ = 2.50 ppm). Molecular weights of poly(ester amide)s were determined using a GPC system (HLC-8220, Tosoh, Tokyo, Japan) at AIST. Poly(methyl methacrylate)s were used as standard substances. A column (TSK gel Super HM-N, Tosoh, Tokyo, Japan) was used with HFIP as an eluent (0.2 mL/min, 40 °C). The *T*_m_ and *T*_g_ of the poly(ester amide)s were measured by DSC (DSC-6220, Seiko Instruments, Tokyo, Japan). TG was performed using TG/DTA-6300 (Seiko Instruments, Tokyo, Japan) in a nitrogen stream.

### 4.3. Synthesis of Ditosylates of Diesterdiamines

GABA (40 mmol), TsOH·H_2_O (40.0 mmol), and diol (20.0 mmol) were added to toluene (70 mL) in a reactor with Dean-Stark equipment (Asahi Glassplant Inc., Arao, Japan). The mixture was stirred and refluxed for 24 h at 130 °C with removing water. The solution was evaporated under reduced pressure and the residue was recrystallized from EtOH. Filtration and evaporation of the solution gave ditosylate monomers as white solids.

gEg-OTs: 89% yield. ^1^H NMR (DMSO-*d*_6_, 500 MHz, r.t.) δ 7.63 (br, 6H, N*H*_3_^+^), 7.48 (d, 4H, *J* = 8 Hz, TsO^−^), 7.12 (d, 4H, *J* = 8 Hz, TsO^−^), 4.24 (s, 4H, OC*H*_2_C*H*_2_O), 2.82 (t, 4H, *J* = 7 Hz, COCH_2_CH_2_C*H*_2_NH_3_^+^), 2.42 (t, 4H, *J* = 7 Hz, COC*H*_2_CH_2_CH_2_NH_3_^+^), 2.29 (s, 6H, TsO^−^), 1.79 (m, 4H, *J* = 7 Hz, COCH_2_C*H*_2_CH_2_NH_3_^+^).

gPg-OTs: 41% yield. ^1^H NMR (DMSO-*d*_6_, 500 MHz, r.t.) δ 7.73 (br, 6H, N*H*_3_^+^), 7.48 (d, 4H, *J* = 8 Hz, TsO^−^), 7.12 (d, 4H, *J* = 8 Hz, TsO^−^), 4.07 (t, 4H, *J* = 6 Hz, OC*H*_2_CH_2_C*H*_2_O), 2.82 (t, 4H, *J* = 7 Hz, COCH_2_CH_2_C*H*_2_NH_3_^+^), 2.43 (t, 2H, *J* = 7 Hz, COC*H*_2_CH_2_CH_2_NH_3_^+^), 2.29 (s, 6H, TsO^−^), 1.88 (m, 2H, *J* = 6 Hz, OCH_2_C*H*_2_CH_2_O), 1.76 (m, 4H, *J* = 7 Hz, COCH_2_C*H*_2_CH_2_NH_3_^+^).

gBg-OTs: 81% yield. ^1^H NMR (DMSO-*d*_6_, 500 MHz, r.t.) δ 7.73 (br, 6H, N*H*_3_^+^), 7.48 (d, 4H, *J* = 8 Hz, TsO^−^), 7.12 (d, 4H, *J* = 8 Hz, TsO^−^), 4.04 (t, 4H, *J* = 6 Hz, OC*H*_2_CH_2_CH_2_C*H*_2_O), 2.82 (t, 4H, *J* = 7 Hz, COCH_2_CH_2_C*H*_2_NH_3_^+^), 2.43 (t, 2H, *J* = 7 Hz, COC*H*_2_CH_2_CH_2_NH_3_^+^), 2.29 (s, 6H, TsO^−^), 1.76 (m, 4H, *J* = 7 Hz, COCH_2_C*H*_2_CH_2_NH_3_^+^), 1.60 (m, 4H, OCH_2_C*H*_2_C*H*_2_CH_2_O).

### 4.4. Synthesis of Sequential Poly(Ester Amide)s with GABA Unit

A tosylate salt of alkylene bis(γ-aminobutyrate) (gEg-OTs, gPg-OTs, or gBg-OTs) (0.14 mmol) and di(*p*-nitrophenyl) ester (T-NP, N-NP, S-NP, G-NP, or A-NP) (0.14 mmol) were dissolved in DMSO (4 mL). This mixture was heated to 70 °C and Et_3_N (7.0 mmol) was added to the solution. The mixture was stirred for 24 h at 70 °C. Then the resulting mixture was poured into excess EtOAc to precipitate the polymer, which was filtered and dried in vacuum.

The yields and molecular weights of the obtained polymers are summarized in Table 1. ^1^H NMR spectra of the obtained polymers are shown in supporting materials.

### 4.5. Biodegradation Tests of the Poly(Ester Amide)s in Seawater and in Activated Sludge

The poly(ester amide)s were treated by frost shattering to make them into fine powders. Biodegradation lab tests of the poly(ester amide)s in seawater and in activated sludge were evaluated from oxygen consumption using BOD tester (BOD200F, TAITEC, Koshigaya, Japan) according to the previous paper [54]. Seawater was collected from Osaka South Port bay area [10]. A standard activated sludge was provided from the Chemicals Evaluation and Research Institute, Tokyo, Japan. The typical procedure is as follows: polymer specimens (30 mg) were added into 250 mL BOD testing bottle and then seawater (200 mL) or the activated sludge (200 mL) was added. Evolved CO_2_ was removed by Ca(OH)_2_ from the BOD closed system. The biodegradation test was carried out at 27 °C with stirring for 1 month. The observed O_2_ consumption volume was corrected by subtraction to O_2_ consumption volume of control. The theoretical amount of consumed O_2_ (mL) means the total O_2_ that should be consumed on complete degradation of sample. Biodegradation (%) was calculated as follows: 

In the case of poly(gEgT), the theoretical consumption of O_2_ is the following Equation (1), and the biodegradation is estimated by Equation (2):

C_20_H_26_N_2_O_6_ + 26 O_2_ → 20 CO_2_ + 12 H_2_O + 2 HNO_3_ (1)

Biodegradation (%) = [experimentally consumed O_2_ (mL)/theoretical O_2_ (mL)] × 100 (2)

The biodegradation value (%) was average value of duplicate data (*n* = 2).

## 5. Conclusions

We succeeded in synthesizing a series of new poly(ester amide)s containing GABA unit and observed their relatively higher *T*_m_ in comparison with those of typical aliphatic polyesters. Compared with PA4, the processability of these poly(ester amide)s were improved. Aliphatic poly(ester amide)s containing ethylene glycol or 1,3-propanediol units showed moderate biodegradability in seawater. Poly(gEgT) was found to show relatively high biodegradability in seawater among the aromatic poly(ester amide)s. Aliphatic poly(ester amide)s containing ethylene glycol unit also exhibited moderate biodegradability in activated sludge. However, the present synthetic method of the poly(ester amide)s is not desirable for their industrial production and the molecular weights of the products are not high enough for most applications. A better synthetic method is desired for their practical application.

## Figures and Tables

**Table 1:** Synthesis of sequential poly(ester amide)s.

Entry	Polymer	*x*	*R*	Yield	*M*_n_ *^a^*	*M*_w_ *^a^*	*M*_w_/*M*_n_*^a^*
				(%)	(g/mol)	(g/mol)	
1	Poly(gEgT)	2	*p*-phenylene	72	2600	4100	1.6
2	Poly(gPgT)	3	*p*-phenylene	92	6500	13,800	2.1
3	Poly(gBgT)	4	*p*-phenylene	80	4900	8300	1.7
4	Poly(gEgN)	2	2,5-naphthalenediyl	92	5000	9900	2
5	Poly(gPgN)	3	2,5-naphthalenediyl	97	5500	12,400	2.2
6	Poly(gBgN)	4	2,5-naphthalenediyl	96	4900	8800	1.8
7	Poly(gEgS)	2	(CH_2_)_2_	86	6100	12,400	2
8	Poly(gPgS)	3	(CH_2_)_2_	83	5700	10,700	1.9
9	Poly(gBgS)	4	(CH_2_)_2_	79	7600	15,300	2
10	Poly(gEgGl)	2	(CH_2_)_3_	77	4100	10,500	2.6
11	Poly(gPgGl)	3	(CH_2_)_3_	88	6700	17,800	2.7
12	Poly(gBgGl)	4	(CH_2_)_3_	83	5700	14,900	2.6
13	Poly(gEgA)	2	(CH_2_)_4_	83	4400	11,000	2.5
14	Poly(gPgA)	3	(CH_2_)_4_	83	7300	18,700	2.5
15	Poly(gBgA)	4	(CH_2_)_4_	78	6600	15,500	2.3

*^a^* Determined by GPC calibrated with PMMA standard in HFIP.

**Table 2:** Thermal properties of the sequential poly(ester amide)s.

Entry	Polymer	*T*_g_ *^a^*	*T*_m_ *^a^*	Δ*H*_m_*^a^*	*T*_d5_ *^b^*	*T*_d10_ *^b^*
		(°C)	(°C)	(J/g)	(°C)	(°C)
1	Poly(gEgT)	103	212, 238	9.6, 11.0	302	320
2	Poly(gPgT)	75	200, 218	5.6, 8.6	276	294
3	Poly(gBgT)	72	188, 207	13.4, 5.2	268	280
4	Poly(gEgN)	84	229, 238	10.6	290	300
5	Poly(gPgN)	90	218, 232	5.6, 6.2	297	311
6	Poly(gBgN)	71	217, 236	14.2	300	315
7	Poly(gEgS)	71	165, 181	17	245	274
8	Poly(gPgS)	84	146, 166	11.0, 8.2	259	277
9	Poly(gBgS)	79	152, 172	11.1, 7.9	264	278
10	Poly(gEgGl)	78	112, 133	1.2, 11.0	269	284
11	Poly(gPgGl)	74	121, 140	13.1, 0.6	269	281
12	Poly(gBgGl)	77	111, 128	12.7, 1.1	271	289
13	Poly(gEgA)	75	162	19.2	279	292
14	Poly(gPgA)	82	140, 151, 162	18.4, 3.3	295	310
15	Poly(gBgA)	79	148, 162	15.0, 3.7	279	290
	PET [47]	81	240–260		396	
	PA4 [47,48]		240–260		236	
	PBS [47]		113			

*^a^* Determined by DSC; *^b^* Determined by TG/DTA.

## Supplementary Materials

Supplementary materials can be found at https://www.mdpi.com/1422-0067/21/10/3674/s1.

## Abbreviations

GABA	γ-Aminobutyric acid
PA4	Polyamide 4
PHA	Poly(3-hydroxyalkanoate)
PLLA	Poly(L-lactic acid)
PGA	Poly(glycolic acid)
PCL	Poly(ε-caprolactone)
PBS	Poly(butylene succinate)
PET	Poly(butylene succinate)
*T*_m_	Melting temperature
*T*_g_	Glass-transition temperature
TsOH·H_2_O	*p*-Toluenesulfonic acid monohydrate
gEg-OTs	Ditosylate of ethylene bis(γ-aminobutyrate)
gPg-OTs	Ditosylate of trimethylene bis(γ-aminobutyrate)
gBg-OTs	Ditosylate of butylene bis(γ-aminobutyrate)
T-NP	Bis(*p*-nitrophenyl) terephthalate
N-NP	Bis(*p*-nitrophenyl) 2.6-naphthalenedicarboxylate
S-NP	Bis(*p*-nitrophenyl) succinate
Gl-NP	Bis(*p*-nitrophenyl) glutarate
A-NP	Bis(*p*-nitrophenyl) adipate
DMSO	Dimethyl sulfoxide
THF	Tetrahydrofuran
HFIP	1,1,1,3,3,3-Hexafluoro-2-propanol
GPC	Gel permeation chromatography
PMMA	Poly(methyl methacrylate)
*M*_n_	Number-averaged molecular weight
*M*_w_	Weight-averaged molecular weight
Poly(gEgT)	Poly((ethylene bis(γ-aminobutyrate)) terephthalate)
Poly(gPgT)	Poly((trimethylene bis(γ-aminobutyrate)) terephthalate)
Poly(gBgT)	Poly((butylene bis(γ-aminobutyrate)) terephthalate)
Poly(gEgN)	Poly((ethylene bis(γ-aminobutyrate)) 2.6-naphthalenedicarboxylate)
Poly(gPgN)	Poly((trimethylene bis(γ-aminobutyrate)) 2.6-naphthalenedicarboxylate)
Poly(gBgN)	Poly((butylene bis(γ-aminobutyrate)) 2.6-naphthalenedicarboxylate)
Poly(gEgS)	Poly((ethylene bis(γ-aminobutyrate)) succinate)
Poly(gPgS)	Poly((trimethylene bis(γ-aminobutyrate)) succinate)
Poly(gBgS)	Poly((butylene bis(γ-aminobutyrate)) succinate)
Poly(gEgGl)	Poly((ethylene bis(γ-aminobutyrate)) glutarate)
Poly(gPgGl)	Poly((trimethylene bis(γ-aminobutyrate)) glutarate)
Poly(gBgGl)	Poly((butylene bis(γ-aminobutyrate)) glutarate)
Poly(gEgA)	Poly((ethylene bis(γ-aminobutyrate)) adipate)
Poly(gPgA)	Poly((trimethylene bis(γ-aminobutyrate)) adipate)
Poly(gBgA)	Poly((butylene bis(γ-aminobutyrate)) adipate)
DSC	Differential scanning calorimetry
TG	Thermogravimetric analysis
*T*_d5_	Degradation temperatures at 5% weight loss
*T*_d10_	Degradation temperatures at 10% weight loss
PHB	Poly(3-hydroxybutyrate)
PES	Poly(ethylene succinate)
AIST	National Institute of Advanced Industrial Science and Technology
