# Supplementary material for: Synthesis, Properties, and Biodegradation of Sequential Poly(Ester Amide)s Containing γ-Aminobutyric Acid
Source: Int J Mol Sci. 2020 May 23;21(10):3674. doi: 10.3390/ijms21103674 (PMC7279392; doi:10.3390/ijms21103674)
Supplement: Supplementary file 1 [file ijms-21-03674-s001.pdf]

Supplementary material

Synthesis, properties, and biodegradation of periodic copoly(ester amide)s containing γ-aminobutyric acid

Yuushou Nakayama [1,*], Kazumasa Watanabe [1], Ryo Tanaka [1], Takeshi Shiono [1], Norioki Kawasaki [2], Naoko Yamano [2] and Atsuyoshi Nakayama [2]

[1] Department of Applied Chemistry, Graduate School of Engineering, Hiroshima University, 1-4-1 Kagamiyama, Higashi-Hiroshima, Hiroshima 739-8527, Japan

[2] Biomedical Research Institute, National Institute of Advanced Industrial Science and Technology (AIST), Osaka, 563-8577, Japan

Contents

1. ^{1}H NMRspectra of the copoly(ester-amide)s. 2

2. DSC curves of the copoly(ester-amide)s. 11

3. TG/DTA curves of the copoly(ester-amide)s. 12

1. ^{1}H and ^{13}C NMR spectra of the products.

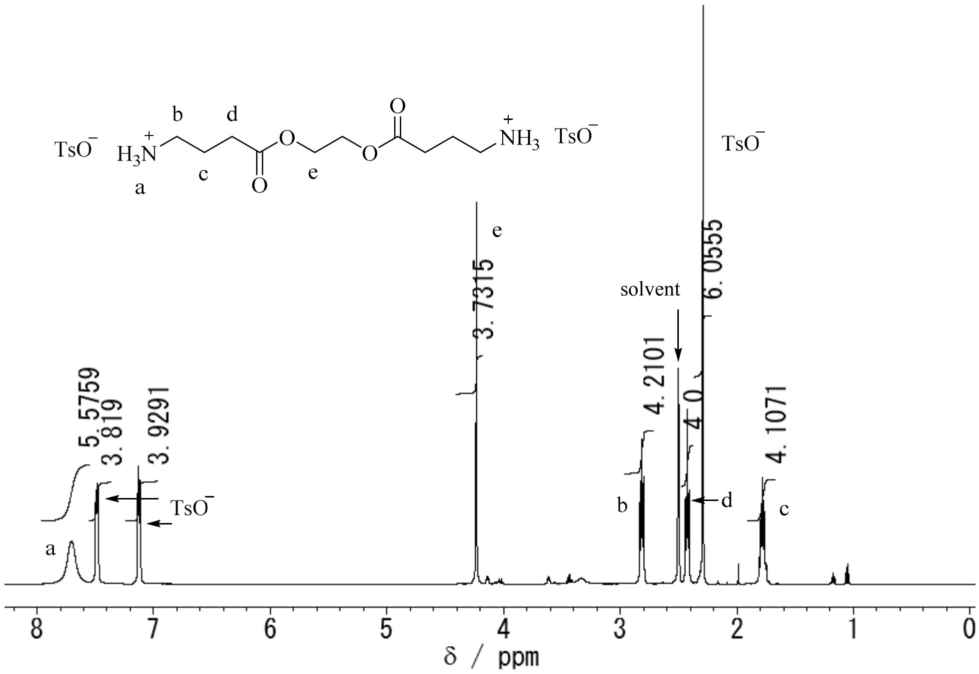

Figure S1. ^{1}H NMR spectrum of gEg-OTs (DMSO-d_6, 500 MHz, r.t.)

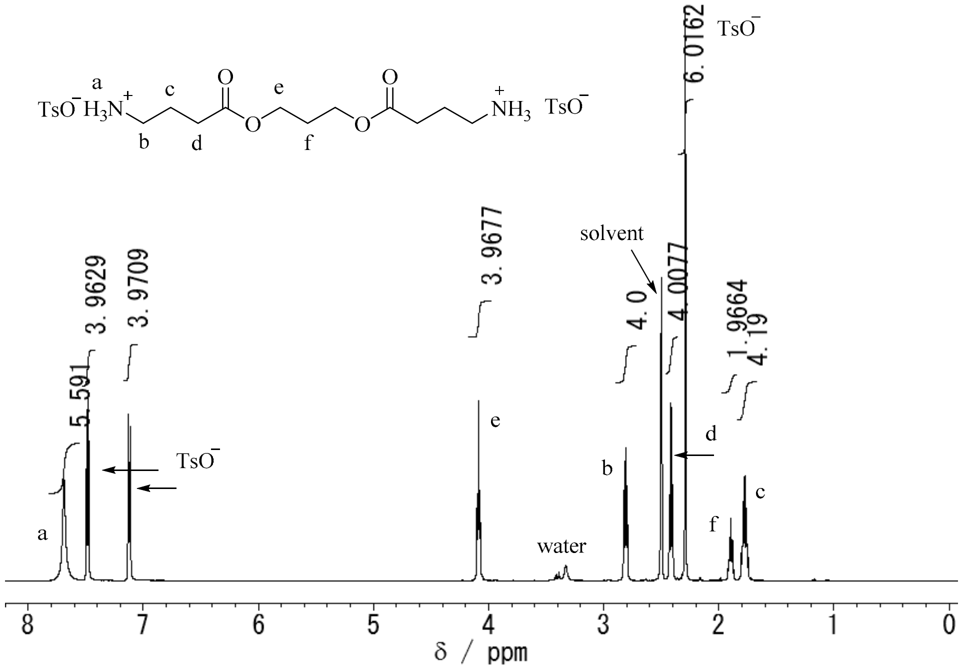

Figure S2. ^{1}H NMR spectrum of gPg-OTs (DMSO-d_6, 500 MHz, r.t.)

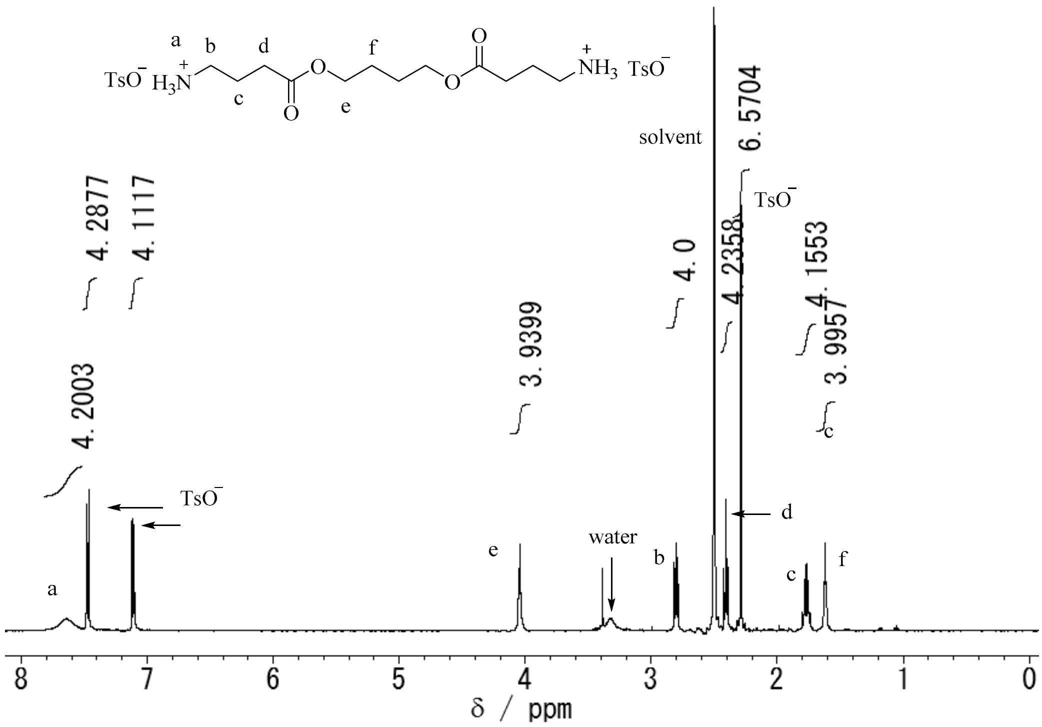

Figure S3. ^{1}H NMR spectrum of gBg-OTs (DMSO-d6, 500 MHz, r.t.)

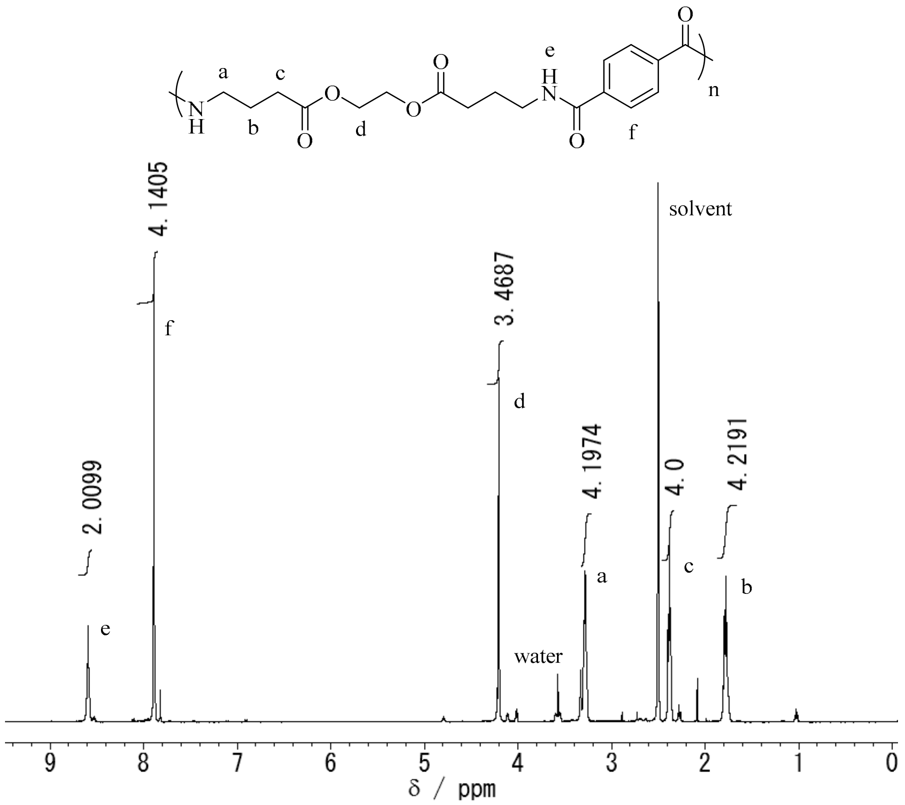

Figure S4. ^{1}H NMR spectrum of poly(gEgT) (DMSO-d6, 500 MHz, r.t.).

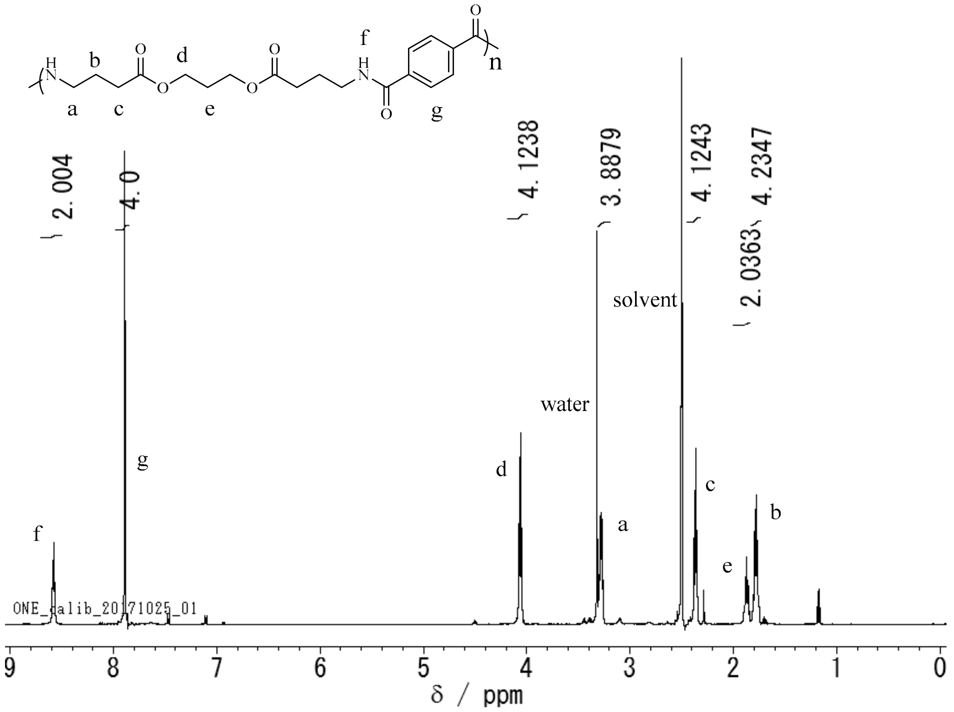

Figure S5. ^{1}H NMR spectrum of poly(gPgT) (DMSO-d_6, 500 MHz, r.t.).

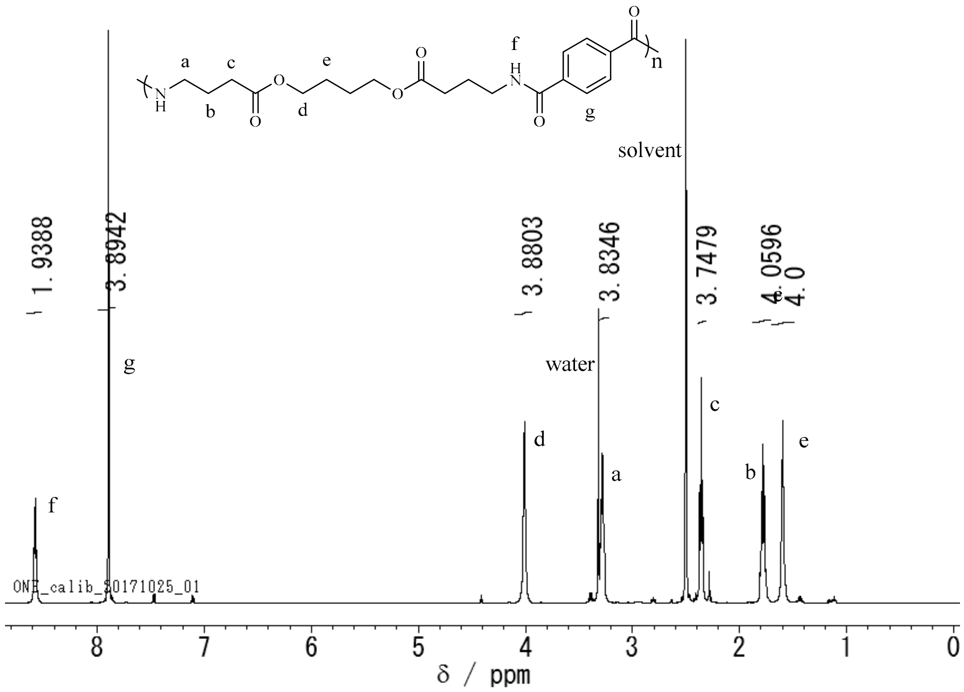

Figure S6. ^{1}H NMR spectrum of poly(gBgT) (DMSO-d_6, 500 MHz, r.t.).

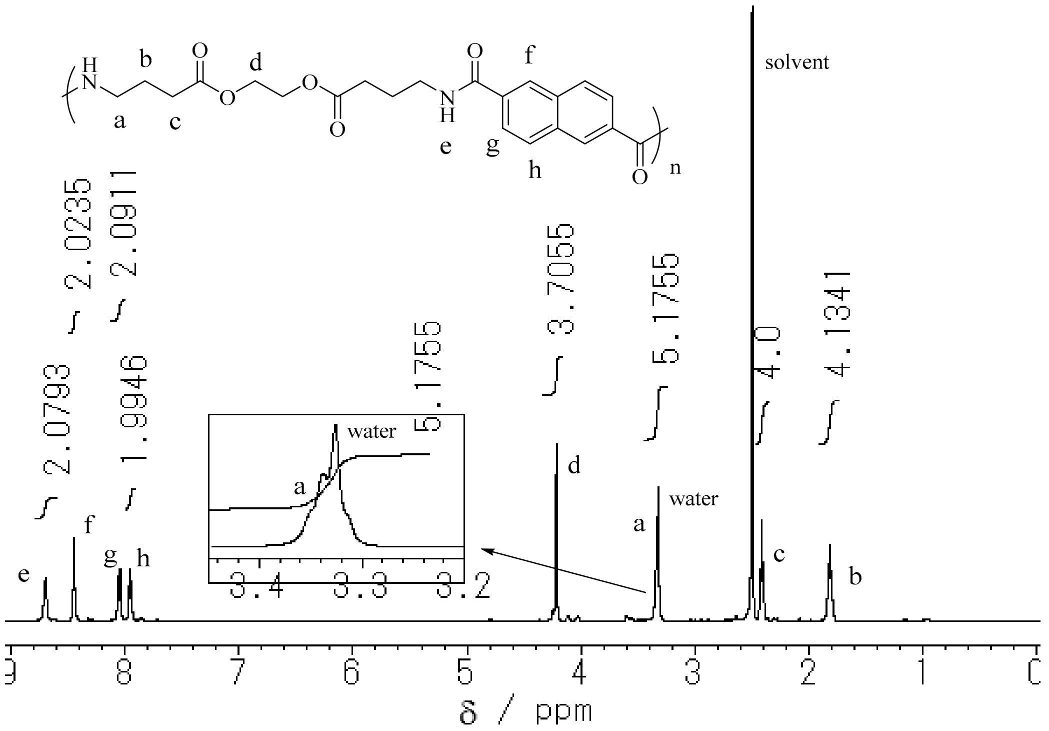

Figure S7. ^{1}H NMR spectrum of poly(gEgN) (DMSO-d$_6$, 500 MHz, r.t.).

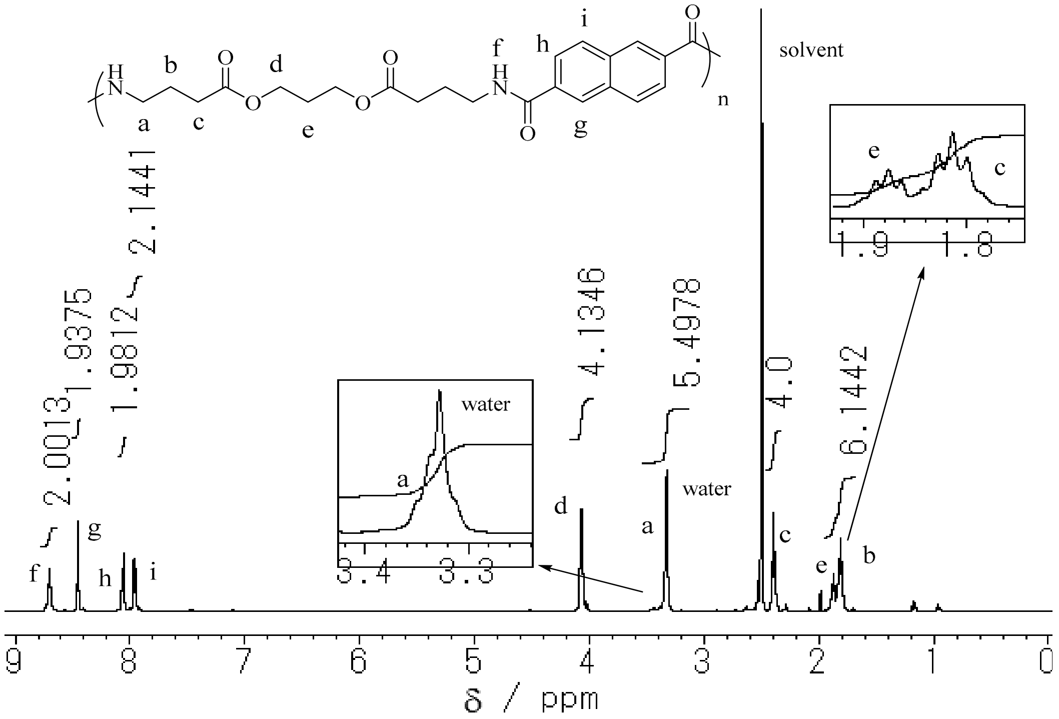

Figure S8. ^{1}H NMR spectrum of poly(gPgN) (DMSO-d$_6$, 500 MHz, r.t.).

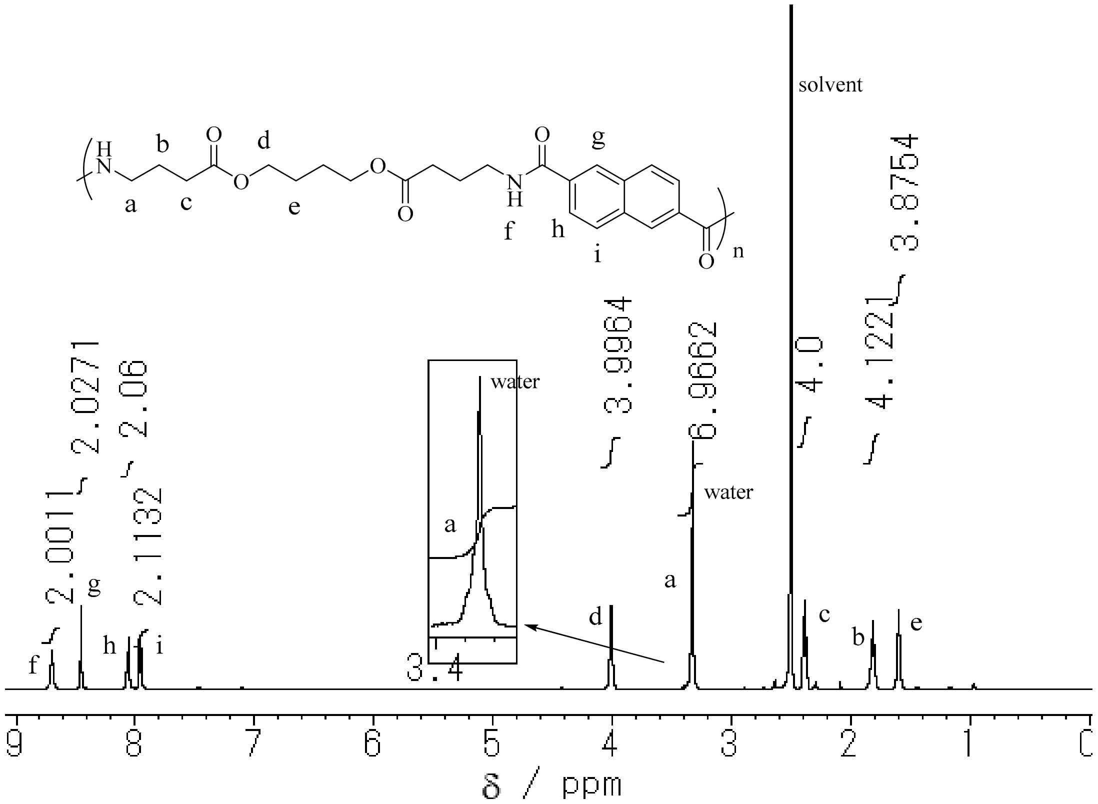

Figure S9. ^{1}H NMR spectrum of poly(gBgN) (DMSO-d_6, 500 MHz, r.t.).

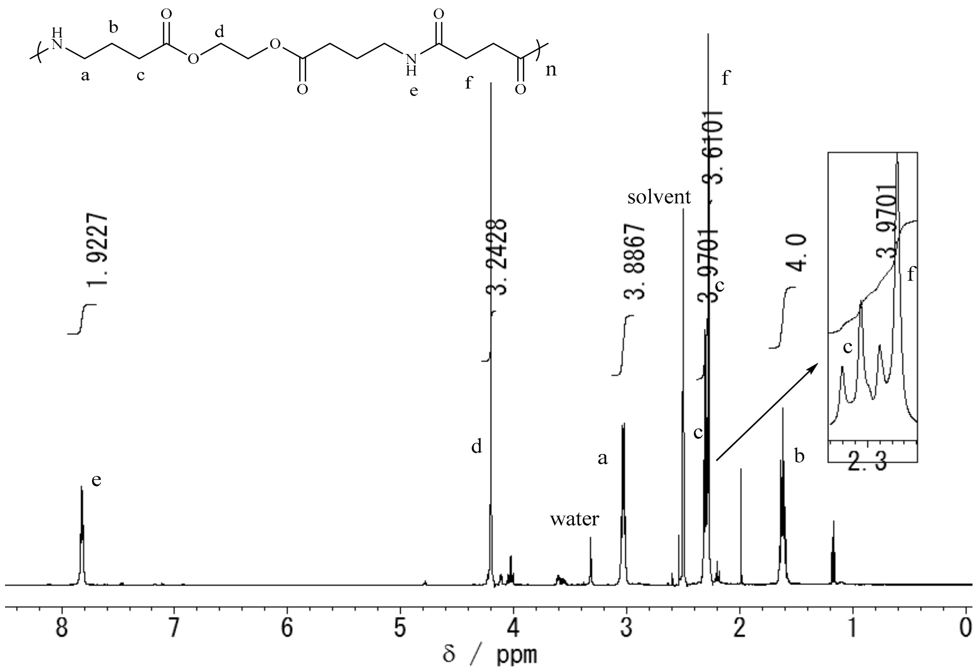

Figure S10. ^{1}H NMR spectrum of poly(gEgS) (DMSO-d_6, 500 MHz, r.t.).

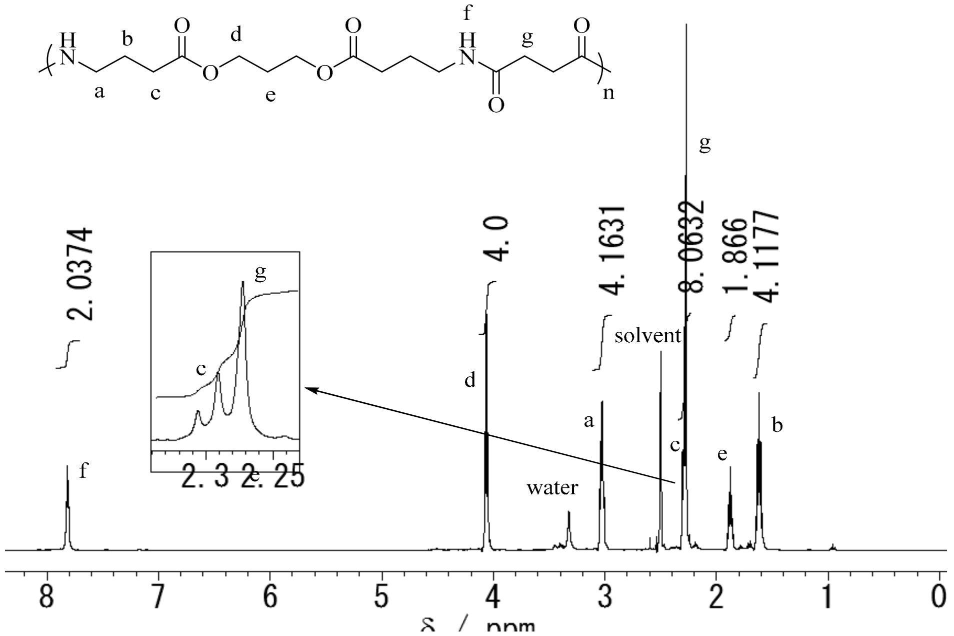

Figure S11. ^{1}H NMR spectrum of poly(gPgS) (DMSO-d6, 500 MHz, r.t.).

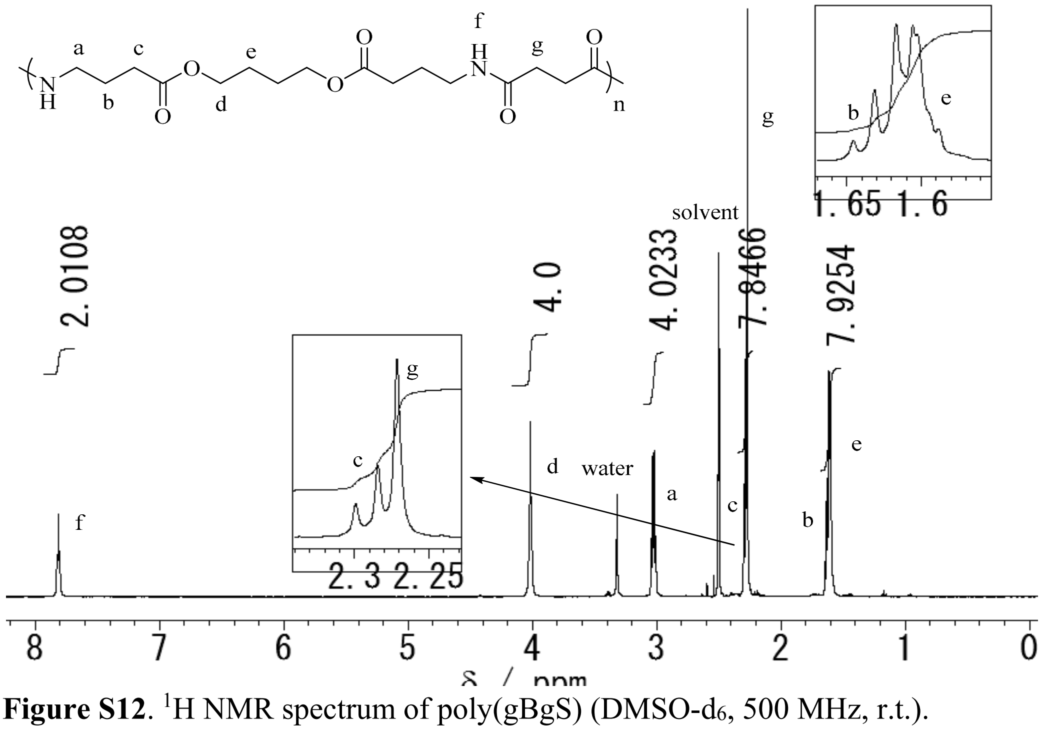

Figure S12. ^{1}H NMR spectrum of poly(gBgS) (DMSO-d6, 500 MHz, r.t.).

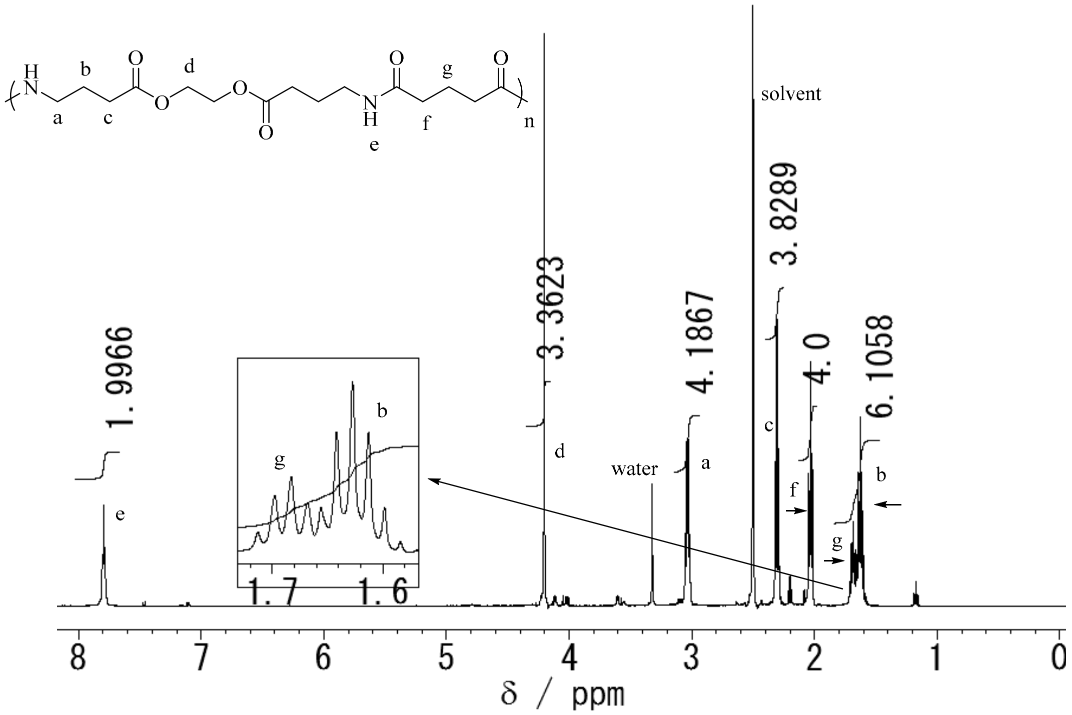

Figure S13. ^{1}H NMR spectrum of poly(gEgGl) (DMSO-d_6, 500 MHz, r.t.).

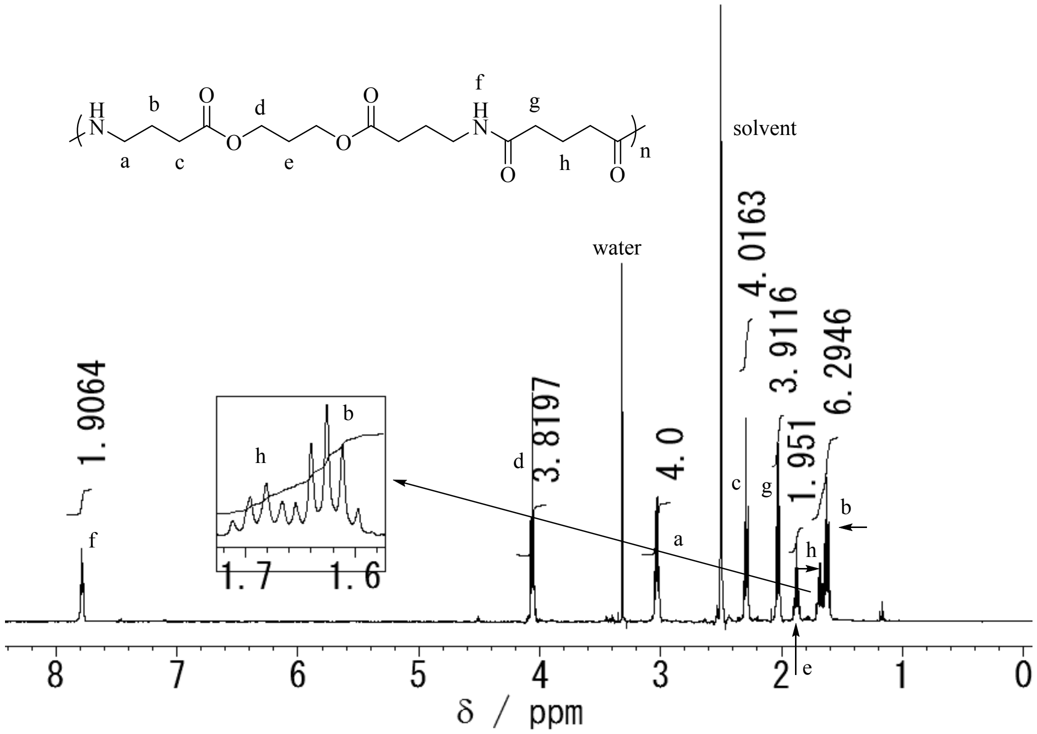

Figure S14. ^{1}H NMR spectrum of poly(gPgGl) (DMSO-d_6, 500 MHz, r.t.).

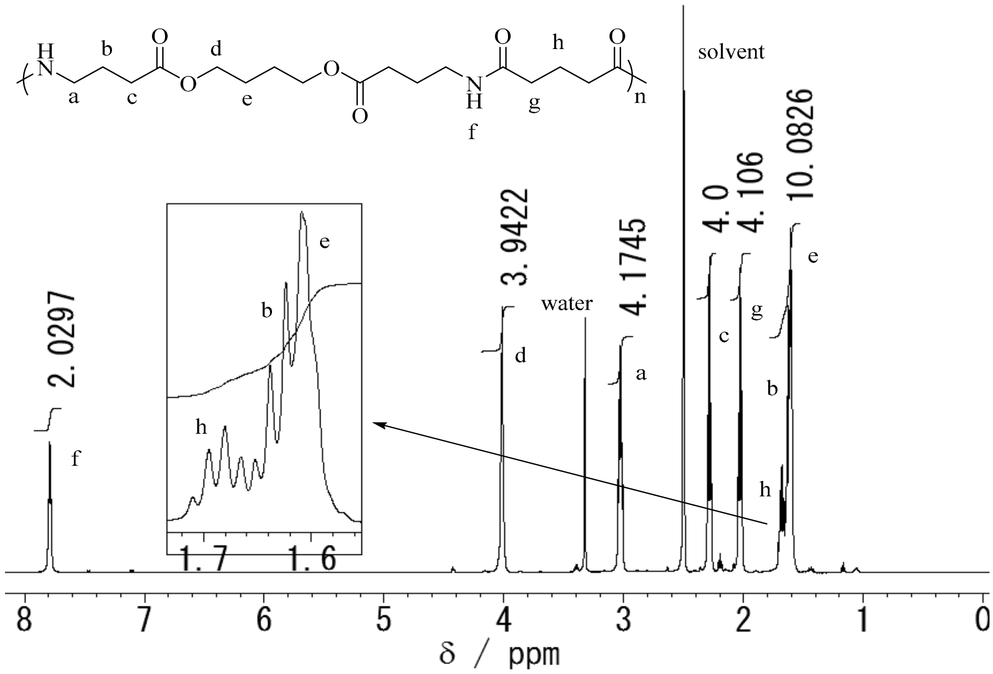

Figure S15. ^{1}H NMR spectrum of poly(gBgGl) (DMSO-d6, 500 MHz, r.t.).

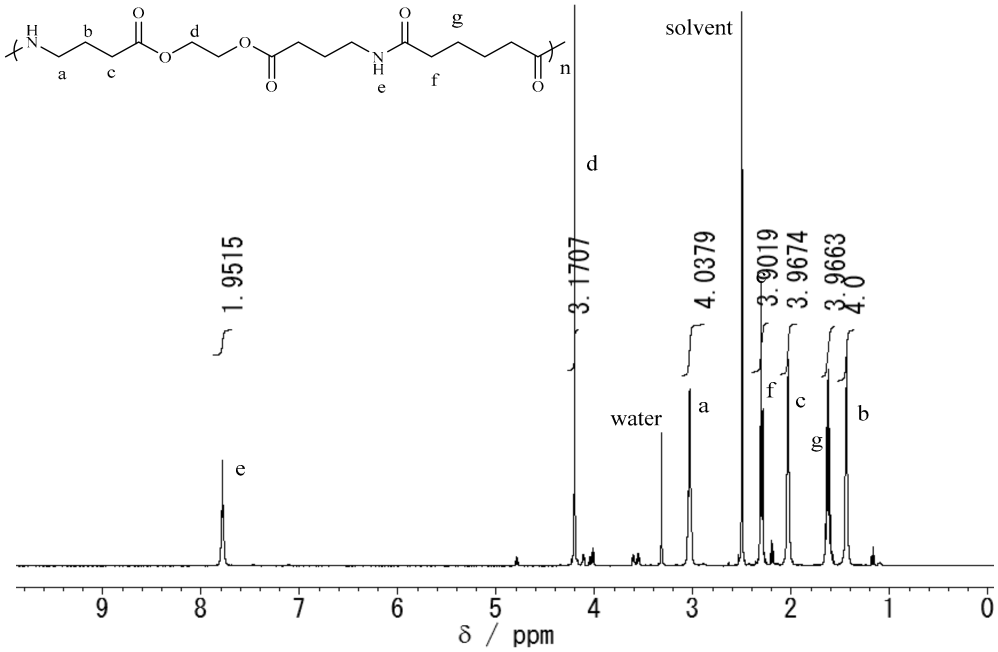

Figure S16. ^{1}H NMR spectrum of poly(gEgA) (DMSO-d6, 500 MHz, r.t.).

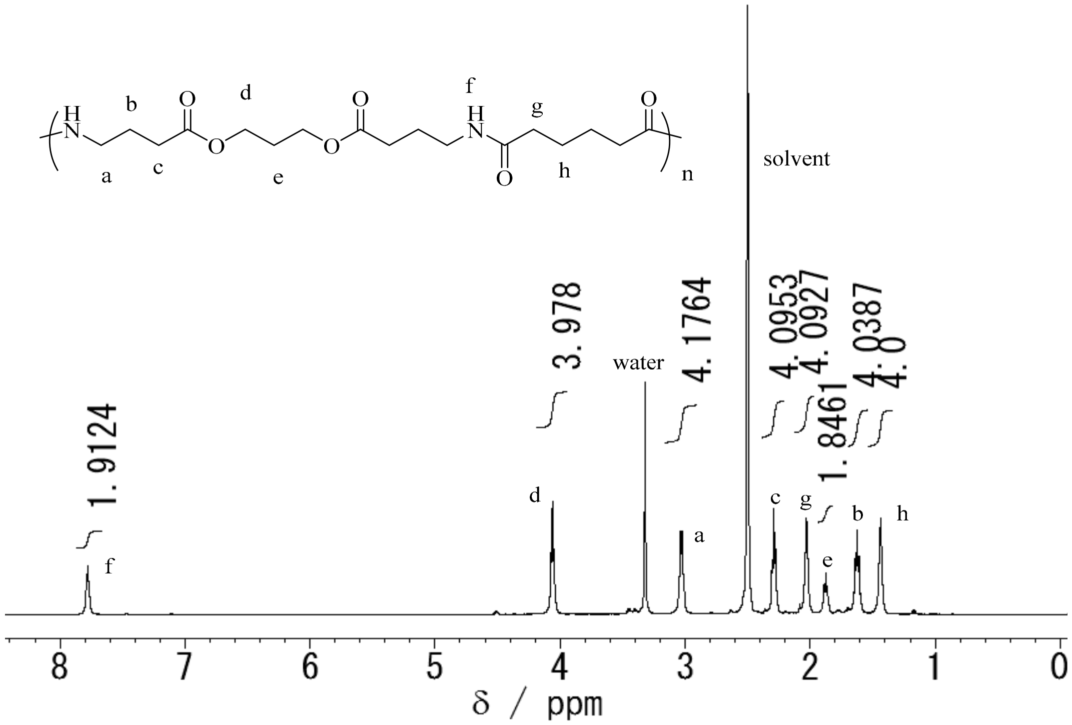

Figure S17. ^{1}H NMR spectrum of poly(gPgA) (DMSO-d_6, 500 MHz, r.t.).

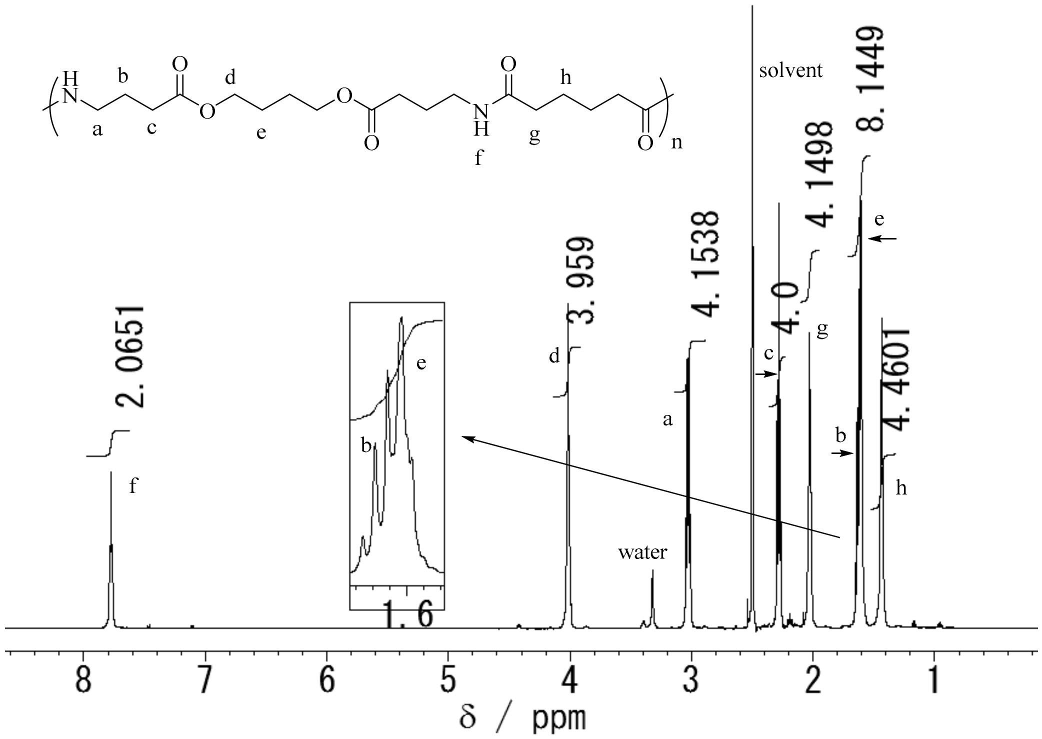

Figure S18. ^{1}H NMR spectrum of poly(gBgA) (DMSO-d_6, 500 MHz, r.t.).

2. DSC Curves of the periodic copoly(ester-amide)s.

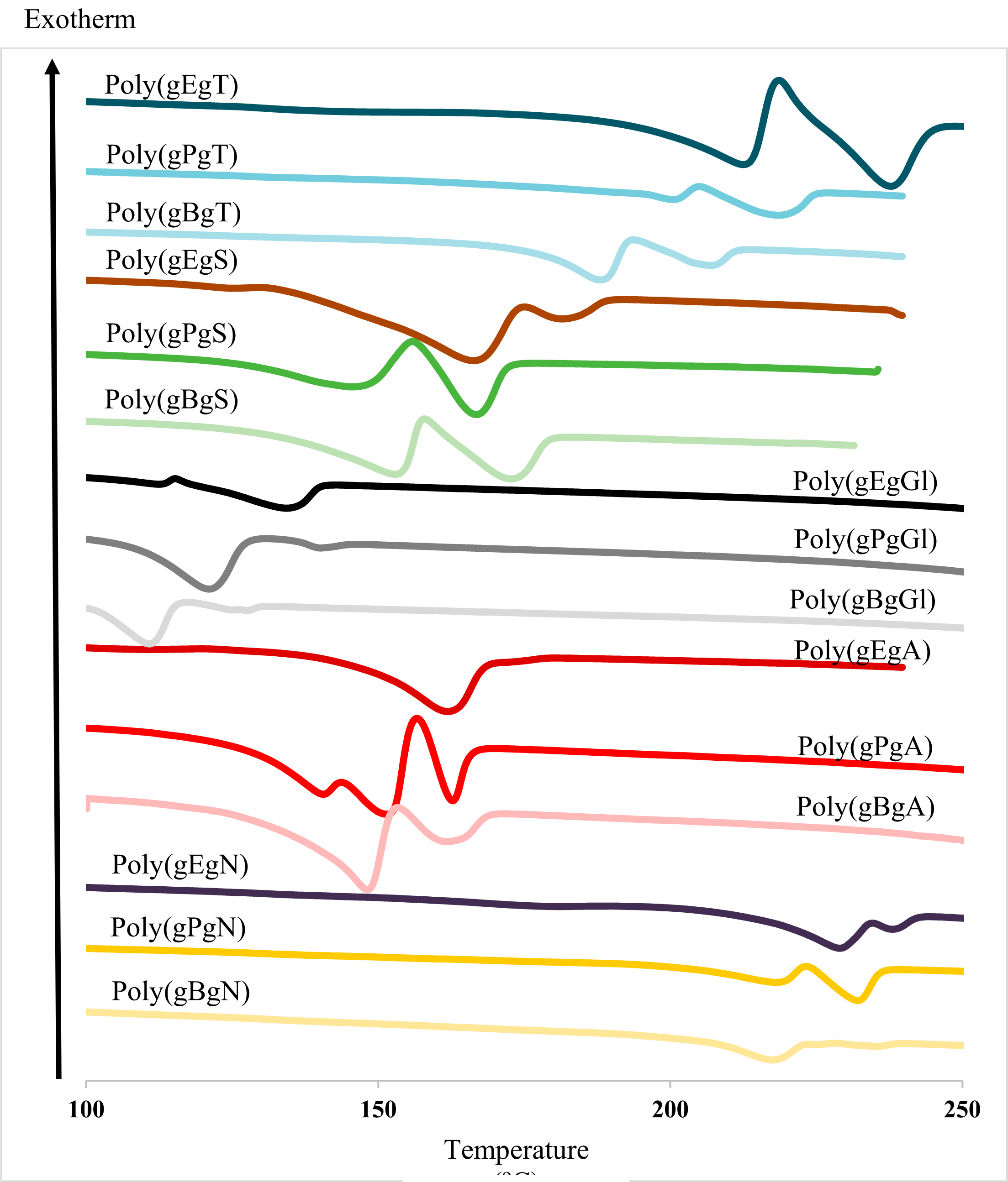

Figure S19. The observed melting transitions in DSC Curves of the periodic copoly(ester-amide)s.

3. TG/DTA curves of the copoly(ester-amide)s.

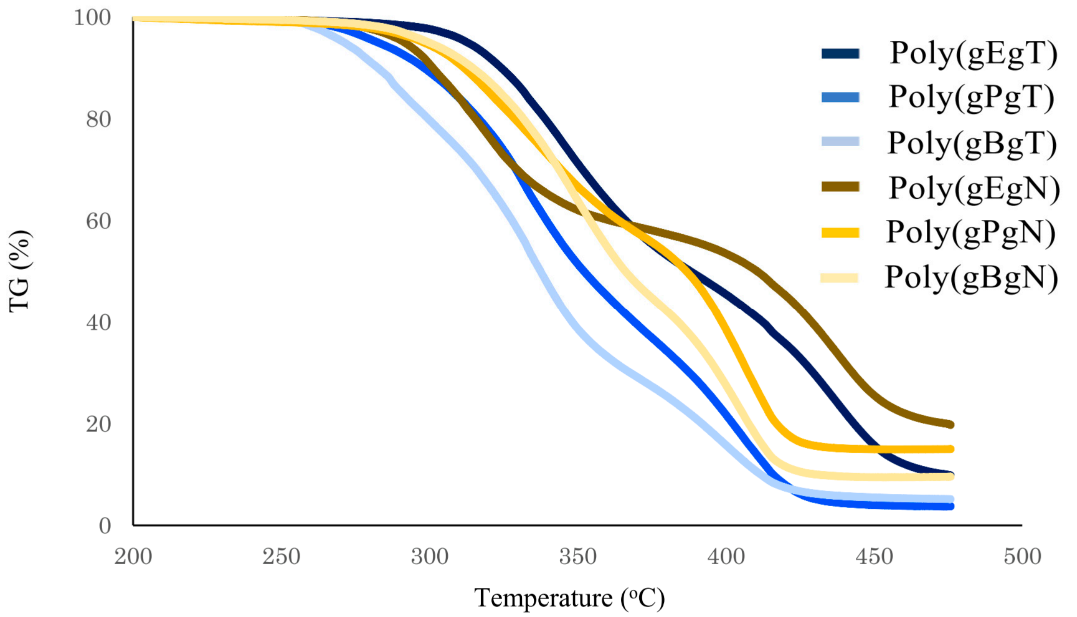

Figure S20. TG/DTA curves of the aromatic copoly(ester amide)s with terephthalate or naphthalenedicarboxylate units.

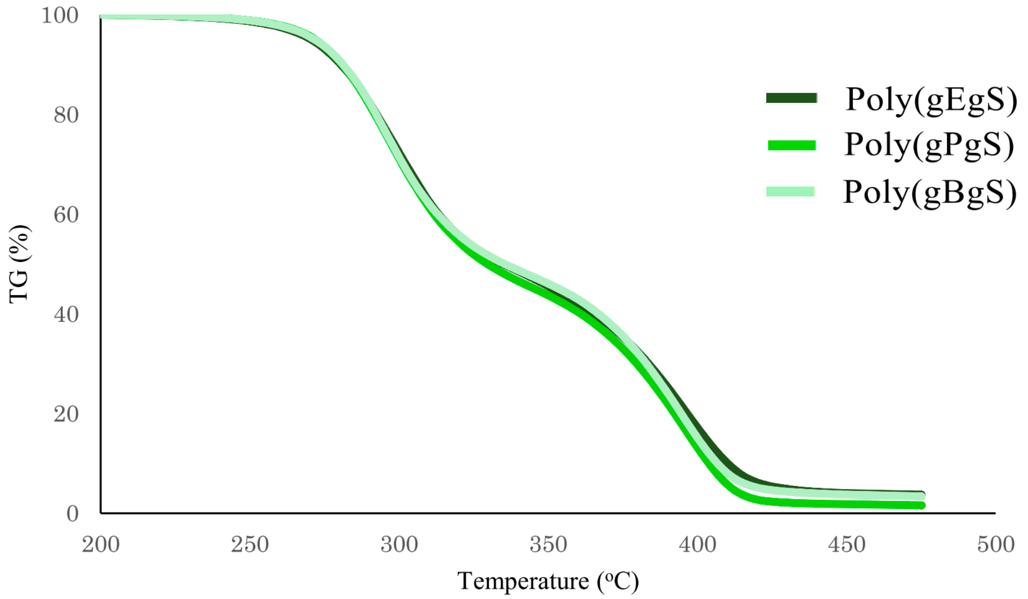

Figure S21. TG/DTA curves of the copoly(ester amide)s with succinate units.

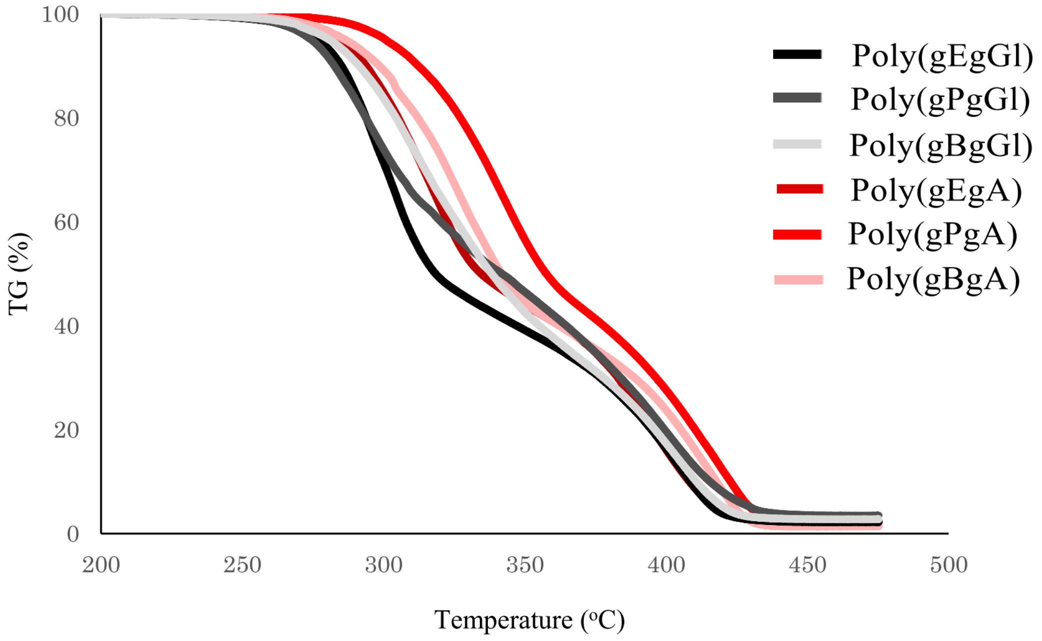

Figure S22. TG/DTA curves of the copoly(ester amide)s with glutarate or adipate units.
